# Past efforts in determining suitable normalization methods for multi-criteria decision-making: A short survey

**DOI:** 10.3389/fdata.2022.990699

**Published:** 2022-08-18

**Authors:** Anath Rau Krishnan

**Affiliations:** Labuan Faculty of International Finance, Universiti Malaysia Sabah, Labuan, Malaysia

**Keywords:** decision criteria, decision matrix, incommensurable data, multi-criteria decision-making, normalization

## Abstract

The use of a multi-criteria decision-making (MCDM) technique mostly begins with normalizing the incommensurable data values in the decision matrix. Numerous normalization methods are available in the literature and applying different normalization methods to an MCDM technique is proven to deliver varying results. As such, selecting suitable normalization methods for an MCDM technique has emerged as an intriguing research topic, especially with the advent of big data. Several efforts have been made to compare the suitability of various normalization methods, but regrettably, no paper provides an updated review of these crucial efforts. This study, therefore, aimed to trace articles reporting such efforts and review them based on the following three perspectives: (1) the normalization methods considered, (2) the MCDM methods considered, and (3) the comparison metrics used to determine the suitable normalization methods. The relevant articles were extracted with the aid of Google Scholar using the keywords of “normalization” and “MCDM,” and Tableau software was used to analyze further the data gathered through the articles. A total of five limitations were uncovered based on the current state of literature, and potential future works to address those limitations were offered. This paper is the first to compile and review the previous investigations that compared and determined the ideal normalization methods for an MCDM technique.

## Introduction

A multi-criteria decision-making (MCDM) technique allows the decision-makers to systematically evaluate the alternatives under consideration based on a predefined set of decision criteria (Talmor, 2021). The technique usually synthesizes the data values of every alternative across different criteria into an aggregated value. Based on these aggregated values, the alternatives are then ranked from a most preferred to a least preferred (Krishnan et al., 2017). Although a wide range of MCDM techniques is available, it is interesting to note that the core input for all these techniques is the decision matrix that encapsulates the data values of alternatives across the decision criteria (Ceballos et al., 2017).

Assume an MCDM problem in which *a*_*i*_ = {*a*_1_, *a*_2_, …, *a*_*m*_} represents the set of alternatives under consideration and *c*_*j*_ = {*c*_1_, *c*_2_, …, *c*_*n*_} represents the set of decision criteria. The general form of the decision matrix can then be expressed as in (1), where *x*_*mn*_ denotes the data value of alternative *m* over criterion *n* (Krishnan et al., 2021).


(1) $$\begin{array}{r} \begin{array}{ccccc} Alternatives/criteria & c_{1} & c_{2} & \ldots & c_{n}\\ a_{1} & x_{11} & x_{12} & \ldots & x_{1n}\\ a_{2} & x_{21} & x_{22} & \ldots & x_{2n}\\ \vdots & \vdots & \vdots & \vdots & \vdots \\ a_{m} & x_{m1} & x_{m2} & \ldots & x_{mn} \end{array} \end{array}$$

The criteria in a decision matrix usually carry different units of measurement (Kosareva et al., 2018). For instance, for a drone selection problem, maximum speed, camera quality, battery power, and the price are measured in meter per second, megapixels, milliampere-hour, and dollars, respectively. As such, the data values in the decision matrix should be converted into a commensurable unit to allow the decision-makers to make a logical data comparison across different criteria (Wen et al., 2020). Indeed, the use of many MCDM methods begins with a procedure of transforming the data in the decision matrix into a standard unit. This procedure is mostly known as normalization. The procedure eliminates unit differences and converts the data values across all criteria to a specific range, such as 0 to 1 (Budiman et al., 2021).

### Motivation

There exist numerous normalization methods in the literature, e.g., sum method, maximum-minimum method, logarithmic method, and vector method (Rodríguez et al., 2021). Associating different normalization methods with an MCDM method could deliver diverging results (Zolfani et al., 2020; Mukhametzyanov, 2021). Therefore, choosing the ideal normalization methods for an MCDM method has emerged as an interesting topic of investigation, especially with the rise of big data. Various efforts were made in the past to examine the suitability of a normalization method. Unfortunately, there is no paper that provides an updated review of such past efforts. Review papers on normalization methods do available, but they were not focused on studies comparing and determining the suitable normalization methods for an MCDM technique. A review of such comparative studies is essential to address the following four questions: (1) What normalization methods have mostly been considered in the comparative studies involving an MCDM technique? (2) What are the MCDM techniques that have been examined for their suitable normalization methods? (3) What are the comparison metrics that have been considered in the literature for determining the suitable normalization methods? (4) What are the limitations in the existing comparative studies that can be addressed in future works? These are the questions that triggered the initiation of this study. In short, there is a necessity to review articles comparing the suitability of the various normalization methods, which could help uncover the current developments in the literature and provide indications for future works based on the existing loopholes.

### Contribution statement

From the literature standpoint, this study is the first to trace and review the previous articles comparing and identifying ideal normalization methods for an MCDM technique. 19 relevant research articles were extracted, to be precise. Also, with the aim of offering a more informative review, we examined each extracted article based on three perspectives: (1) the normalization methods considered, (2) the MCDM methods considered, and (3) the comparison metrics used to determine the suitable normalization methods. As such, ten popular normalization methods and eleven comparison metrics were identified. More importantly, based on the existing state of literature, a total of five limitations were exposed and potential future works to address those limitations were suggested.

Meanwhile, from the practical standpoint, our study relays a strong message to decision-makers that the appropriateness of a normalization method must be tested before employing an MCDM technique, so that a convincing decision can be made for the problem at hand. The study also implies that decision-makers can consider synthesizing results from multiple normalization methods to ensure a more compromised decision is reached.

## Methodology

We tracked the relevant literature works using an academic search engine, namely Google Scholar since it remains the most comprehensive source for academic publications (Martín-Martín et al., 2021). The following two keywords, “normalization” and “MCDM,” were used to make our search more focused. We then screened through the abstract section of the extracted articles and retained only the articles that compare the suitability or effect of various normalization methods over MCDM techniques, i.e., comparative studies. Articles that merely provide an overview of the available normalization were discarded. As a result, 19 comparative studies published from 2000 to 2021 were finalized for further review. We compared all these 19 articles based on the following three perspectives: (1) the normalization methods considered, (2) the MCDM methods considered, and (3) the metrics used to determine the ideal normalization methods. Visual analysis was also performed using Tableau software based on the three perspectives to gain more insights into the current state of the literature.

## Results

Table 1 summarizes the finalized articles according to their year of publication, authors, and MCDM technique(s) considered. The normalization methods that were compared in those articles are also mapped in Table 1. The formula of every normalization method identified in Table 1 is made available in Table 2. Note that only dimensionless normalization methods were included in our review since these are the methods that consider whether a criterion is a benefit or cost criterion before normalization—the benefit criterion implies that the higher the value, the better, whereas the cost criterion means that the lower the value, the better. Thus, using a dimensionless method permits a logical aggregation of the values across different types of criteria. Meanwhile, in Table 3, we mapped the articles to the comparison metrics used therein.

**Table 1:** Summary of articles according to MCDM technique and normalization methods.

**No**.	**Year**	**Source**	**MCDM technique**	**N1**	**N2**	**N3**	**N4**	**N5**	**N6**	**N7**	**N8**	**N9**	**N10**
1	2001	Pavličić (2001)	SAW, TOPSIS & ELECTRE		X		X		X				
2	2005	Milani et al. (2005)	TOPSIS	X		X	X		X				
3	2009	Chakraborty and Yeh (2009)	TOPSIS	X	X	X			X				
4	2012	Liao et al. (2012)	TOPSIS	X	X	X			X				
5	2014	Çelen (2014)	TOPSIS	X	X	X			X				
6	2014	Baghla and Bansal (2014)	VIKOR	X	X		X						
7	2014	Lakshmi and Venkatesan (2014)	TOPSIS	X	X	X			X				
8	2014	Chatterjee and Chakraborty (2014)	TOPSIS, PROMETHEE & GRA		X				X	X			X
9	2015	Vafaei et al. (2015)	TOPSIS	X	X				X		X		
10	2016	Vafaei et al. (2016)	AHP	X	X	X			X		X		
11	2017	Mathew et al. (2017)	WASPAS	X	X	X			X		X	X	
12	2018	Vafaei et al. (2018)	SAW	X	X	X			X		X		
13	2019	Palczewski and Sałabun (2019)	PROMETHEE	X	X	X			X		X		
14	2019	Vafaei et al. (2019)	Dynamic Multi-Criteria Decision-Making	X	X	X			X		X		
15	2020	Vafaei et al. (2020)	AHP	X	X	X			X		X		
16	2020	Jafaryeganeh et al. (2020)	ELECTRE -SAW- TOPSIS		X	X			X		X		
17	2021	Polska et al. (2021)	Logic Scoring of Preference (LSP)	X	X	X			X		X		
18	2021	Ersoy (2021)	ROV	X	X	X	X	X	X	X		X	
19	2021	Lahby et al. (2014)	Mahalanobis distance-based ranking algorithm	X	X	X							

*Sum method (N1), maximum-minimum method (N2), maximum method version I (N3), maximum version II (N4), maximum method version III (N5), vector method (N6), non-linear method (N7), logarithmic method (N8), enhanced accuracy method (N9), Jüttler's-Körth's method (N10), and fuzzification-trapezoidal method (N11)*.

**Table 2:** List of formulas.

**Normalization method**	**Formula for benefit criterion**	**Formula for cost criterion**
N1	$r_{ij}=\frac{x_{ij}}{\overset{m}{\underset{i=1}{\sum }}x_{ij}}$	$r_{ij}=\frac{1/x_{ij}}{\overset{m}{\underset{i=1}{\sum }}x_{ij}}$
N2	$r_{ij}=\frac{x_{ij}-\min_{j}\left(x_{ij}\right)}{\max_{j}\left(x_{ij}\right)-\min_{j}\left(x_{ij}\right)}$	$r_{ij}=\frac{\max_{j}\left(x_{ij}\right)-x_{ij}}{\max_{j}\left(x_{ij}\right)-\min_{j}\left(x_{ij}\right)}$
N3	$r_{ij}=\frac{x_{ij}}{\max_{j}\left(x_{ij}\right)}$	$r_{ij}=1-\frac{x_{ij}}{\max_{j}\left(x_{ij}\right)}$
N4	$r_{ij}=\frac{x_{ij}}{\max_{j}\left(x_{ij}\right)}$	$r_{ij}=\frac{\min_{j}\left(x_{ij}\right)}{x_{ij}}$
N5	$r_{ij}=\frac{x_{ij}}{\max_{j}\left(x_{ij}\right)}$	$r_{ij}=1-\frac{x_{ij}-\min_{j}\left(x_{ij}\right)}{\max_{j}\left(x_{ij}\right)}$
N6	$r_{ij}=\frac{x_{ij}}{\sqrt{\overset{m}{\underset{i=1}{\sum }}x_{ij}^{2}}}$	$r_{ij}=1-\frac{x_{ij}}{\sqrt{\overset{m}{\underset{i=1}{\sum }}x_{ij}^{2}}}$
N7	$r_{ij}={\left(\frac{x_{ij}}{\max_{j}\left(x_{ij}\right)}\right)}^{2}$	$r_{ij}={\left(\frac{\min_{j}\left(x_{ij}\right)}{x_{ij}}\right)}^{3}$
N8	$r_{ij}=\frac{\ln\;\left(x_{ij}\right)}{\ln\;\left(\overset{m}{\underset{i=1}{\prod }}x_{ij}\right)}$	$r_{ij}=\frac{1-\frac{\ln\;\left(x_{ij}\right)}{\ln\;\left(\overset{m}{\underset{i=1}{\prod }}x_{ij}\right)}}{m-1}$
N9	$r_{ij}=1-\frac{\max_{j}\left(x_{ij}\right)-x_{ij}}{\overset{m}{\underset{i=1}{\sum }}\left(\max_{j}\left(x_{ij}\right)-x_{ij}\right)}$	$r_{ij}=1-\frac{x_{ij}-\min_{j}\left(x_{ij}\right)}{\overset{m}{\underset{i=1}{\sum }}\left(x_{ij}-\min_{j}\left(x_{ij}\right)\right)}$
N10	$r_{ij}=1-|\frac{\max_{j}\left(x_{ij}\right)-x_{ij}}{\max_{j}\left(x_{ij}\right)}|$	$r_{ij}=1-|\frac{\min_{j}\left(x_{ij}\right)-x_{ij}}{\min_{j}\left(x_{ij}\right)}|$

*x_ij_ = data value of alternative i with respect to criterion j, r_ij_ = normalized value of alternative i with respect to criterion j, $\underset{j}{\max}\left(x_{ij}\right)$ = maximum value of criterion j, and $\underset{j}{\min}\left(x_{ij}\right)$ = minimum value of criterion j*.

**Table 3:** Summary of articles according to the comparison metrics used.

**Source**	**Ranking consistency index (RCI)**	**Average Spearman correlation (ASC)**	**Average Pearson correlation (APC)**	**Standard deviation (SD)**	**Manhattan distance (MD)**	**Euclidean distance (ED)**	**Chebyshev distance (CD)**	**Ranking abnormalities (RA)**	**Percentage of handoffs (POH)**	**Time complexity (TC)**	**Space complexity (SC)**
Pavličić (2001)	Not applicable—These studies only compared the effects of different normalization methods but did not attempt to find the most suitable ones using any of the identified metrics.										
Milani et al. (2005)	
Chakraborty and Yeh (2009)	X										
Liao et al. (2012)	X										
Çelen (2014)											
Baghla and Bansal (2014)								X	X		
Lakshmi and Venkatesan (2014)										X	X
Chatterjee and Chakraborty (2014)		X									
Vafaei et al. (2015)		X									
Vafaei et al. (2016)		X	X								
Mathew et al. (2017)		X									
Vafaei et al. (2018)	X										
Palczewski and Sałabun (2019)											
Vafaei et al. (2019)	X	X		X	X	X	X				
Vafaei et al. (2020)	X		X	X	X	X	X				
Jafaryeganeh et al. (2020)		X									
Polska et al. (2021)	X										
Ersoy (2021)	X	X	X	X	X	X	X				
Lahby et al. (2014)								X	X		

**Figure 1:**
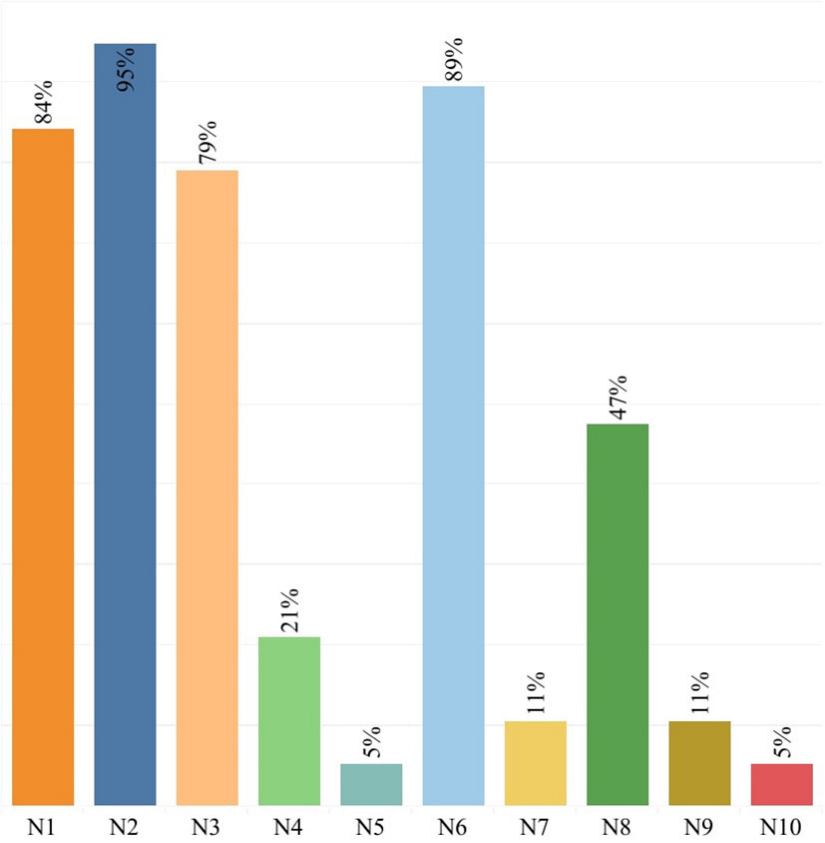
Bar graph showing the percentage of articles vs. normalization methods.

**Figure 2:**
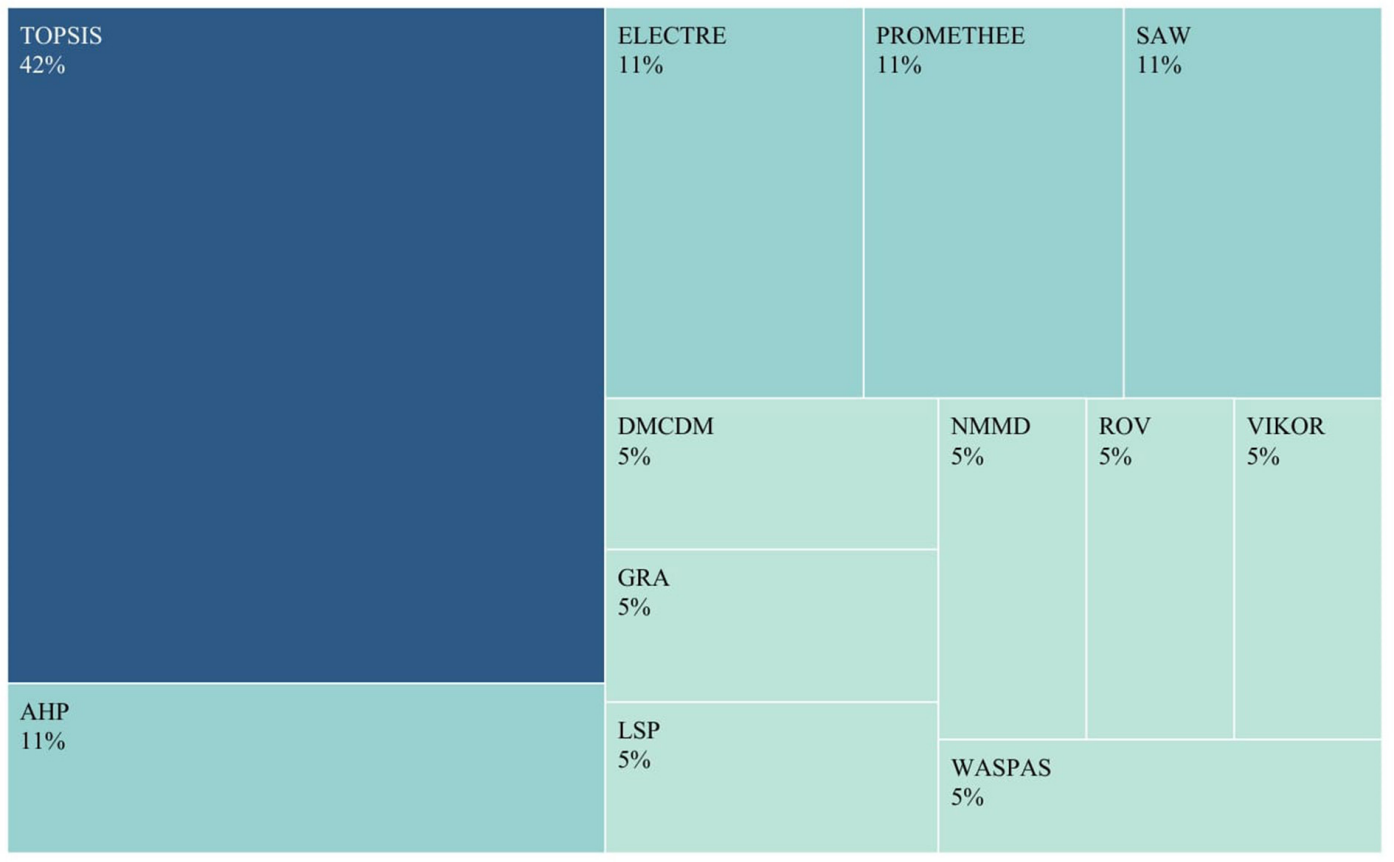
Treemap showing the percentage of articles vs. MCDM techniques.

**Figure 3:**
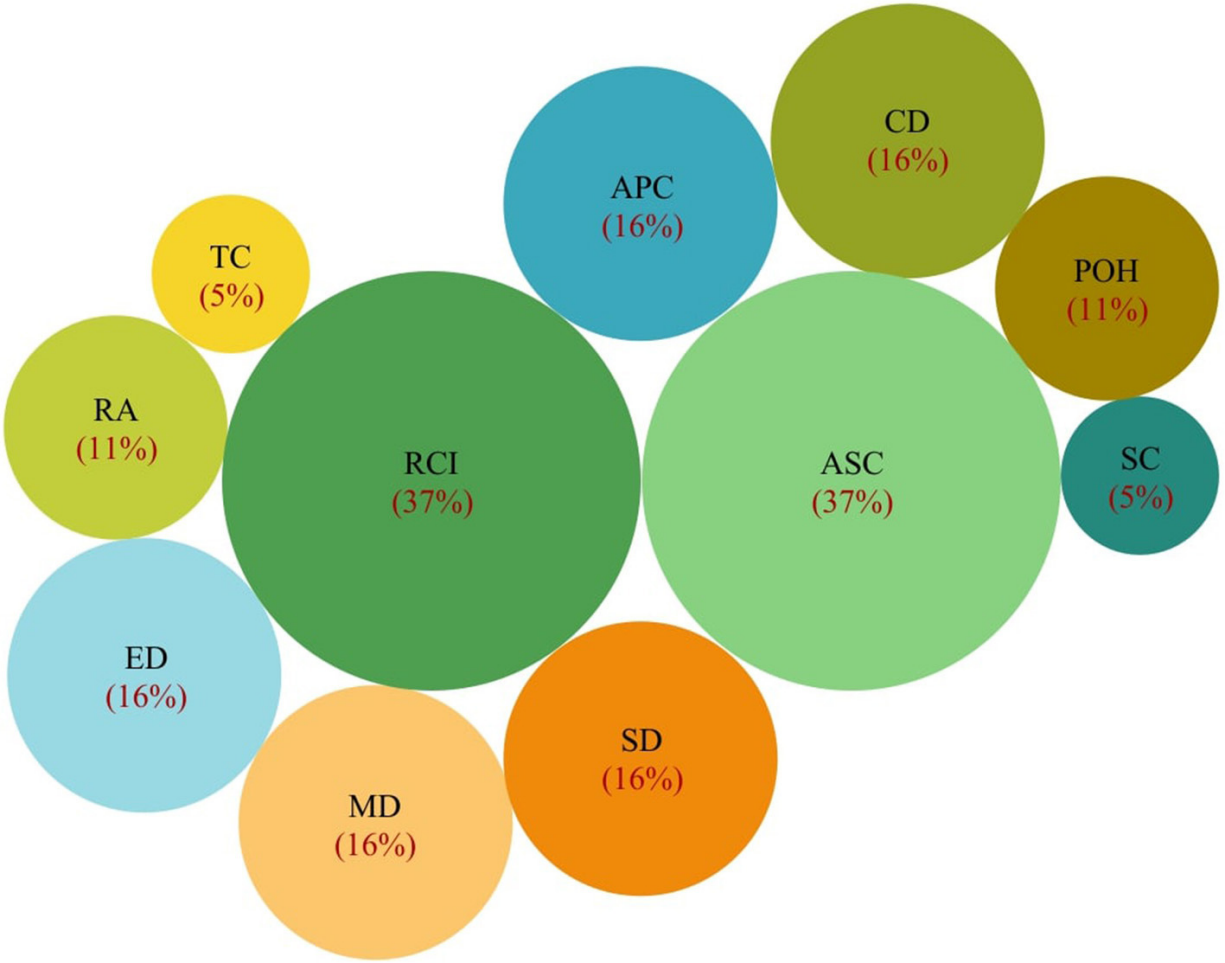
Bubble graph showing the percentage of articles vs. comparison metrics used.

Besides, the results of the visual analysis performed with the aid of Tableau software are depicted in Figures 1–3. Figure 1 is the bar graph that depicts the percentage of articles that considered each identified normalization method. The treemap in Figure 2 shows the percentage of the articles according to each MCDM technique investigated in the past. Lastly, Figure 3 is the bubble graph that reports the percentage of the articles based on each available comparison metric. Interested readers who wish to interact with these figures may access the links provided in the supplementary material section.

## Discussion

Based on the 19 relevant studies traced from the Google Scholar search engine, we identified ten dimensionless normalization methods applicable to MCDM problems. Among all these ten methods, N1, N2, N3, N6, and N8 are the five methods that were mostly compared for their suitability for an MCDM method. However, based on Figure 1, it is obvious that N2 is the topmost normalization method in the literature, where 95% of the existing comparative studies have considered this method. One sure reason for this situation is that N2 is the default normalization method for many MCDM techniques, including VIKOR (Opricovic and Tzeng, 2004), PROMETHEE (Singh et al., 2021) and ELECTRE (Izar-Landeta and Hernandez-Molinar), apart from its advantage in preserving the relationships among the original data values (Kamber and Han, 2012). Note that the default normalization method refers to the normalization method proposed in the original MCDM method, thus not allowing the researchers to disregard it from the comparative studies conducted. The popularity of N2 can also be attributed to its ability to convert the minimum value of a criterion to a 0, the maximum value to a 1, and the rest to a decimal between 0 and 1 (Zach, 2021). However, N2 is very sensitive to the presence of outliers and thus has the risk of producing biased results since it takes into account both the maximum and minimum values (Talukder et al., 2017). Despite its popularity, the majority of comparative studies in Table 1 did not report N2 as the most suitable normalization method, probably sourced by the method's inefficiency in dealing with outliers. Indeed, the test carried out by Vafaei et al. (2015) has identified N2 as the most unfitting method for TOPSIS.

The second most popular normalization method is N6. This finding is unsurprising, given that N6 is the default normalization method for TOPSIS, one of the most extensively investigated MCDM methods in the comparative studies listed in Table 1. Additionally, researchers have expressed a greater interest in N6, possibly because it retains the distribution of the original values even after normalization (Liao and Wu, 2020).

The third and fourth most popular methods are N1 and N3. Eighty-four percentage and 79% of the comparative studies in Table 1 have considered N1 and N3, respectively. The popularity of these two methods can be credited to their computational simplicity. Meanwhile, N8 appears as the fifth most popular method. The method has been gaining growing attention in many real applications mainly because of its appropriateness when the values of the criteria in a decision matrix vary significantly (Zavadskas and Turskis, 2008).

On the other hand, Figure 2 indicates that TOPSIS is the most popular MCDM technique examined for its suitable normalization methods. To be precise, 35% of the studies in Table 1 focused on TOPSIS. The technique has garnered the attention of the majority of studies, most likely due to the numerous advantages it possesses over other MCDM techniques. Some of the advantages of TOPSIS include its simplicity of use, its ability to accommodate all types of criteria (subjective and objective), its rational logic and ease of comprehension by practitioners, its straightforward calculation process, and the ability to visualize the aggregated values of all alternatives on a polyhedron (Shih et al., 2007).

Meanwhile, in the context of comparison metrics used to test the suitability of various normalization methods, we identified a total of eleven such metrics from the existing literature (refer to Table 3). Most of these metrics determine suitability by measuring the consistency degree across the results produced by a method to those produced by other methods. Metrics such as ranking consistency index (RCI) and average Spearmen correlation analyze the consistency in terms of the final alternative ranks resulting from all the normalization methods (Aytekin, 2021). Indeed, these two metrics are reported as the most popular comparison metrics (refer to Figure 3), where 37% of the extracted articles used these metrics.

On the other hand, the average Pearson correlation metric works similar to RCI and Spearmen correlation, but it calculates the consistency across alternative aggregated values, not alternative ranks. Another metric, the standard deviation, is used to measure the variation in the alternative aggregated values resulting from each normalization method. A method with a higher standard deviation is more desired (Ersoy, 2021; Rodríguez et al., 2021) since it indicates the method can better distinguish the performance of alternatives under consideration. Figure 3 shows that about 16% of the existing comparative studies utilized the average Pearson correlation and standard deviation metric.

Figure 3 also reports that previous studies have given significant attention to distance-based metrics, i.e., Manhattan distance, Euclidean distance, and Chebyshev distance. These three metrics quantify the similarity degree of the aggregated values derived through a normalization method with other methods (Trebuna et al., 2014). Like the metrics mentioned above (RCI, average Spearman correlation, average Pearson correlation, and standard deviation), the method with higher scores against these distance-based metrics indicates better suitability (Vafaei et al., 2019).

It is also evident that most metrics in the literature were intended to compare suitability from the result consistency or similarity perspective, except metrics used by Lakshmi and Venkatesan (2014). Unlike others, Lakshmi and Venkatesan compared the normalization methods based on a rare perspective, i.e., the usability perspective. They used two usability metrics, namely the time complexity and space complexity, to compare the suitability of methods such as N1, N2, N3, and N6 over the TOPSIS technique. Finally, they concluded that N1 is the best since it consumes the least time and space than others. This conclusion contradicts the findings of Liao et al. (Liao et al., 2012), who reported N6 as the best normalization method for TOPSIS. It is because Liao et al. conducted the comparative study from the result consistency perspective, not usability. This situation indicates that the comparison metrics used can have an effect on the selection of appropriate normalization methods.

All in all, in light of the current state of literature, it is evident that there exist a few limitations which can be addressed in the future. Following are our five recommendations for future works in response to the limitations we identified through this literature analysis.

### The first limitation and recommendation

The literature review reveals that to this date, no single study has concurrently considered all the ten normalization methods identified in Table 1, for an MCDM technique. Therefore, the conclusiveness of the prior results can be argued. For instance, Baghla and Bansal (Baghla and Bansal, 2014) only considered N1, N2, N3, N6, N8, and N9, to examine the suitability of these six methods for the WASPAS technique. Their investigation concluded N2 and N8 as the most and least preferred method for WASPAS, respectively. However, they could have ended up with a different conclusion if a more comprehensive list of normalization methods had been considered. We, therefore, recommend that any future efforts that aim to identify suitable normalization methods for an MCDM should take into account all the ten methods in Table 1 or more to ensure a definitive conclusion can be reached. However, certain methods can be dropped in such future works subject to the MCDM technique involved. It is because some normalization methods may not be viable with certain MCDM methods. For example, N8 is claimed to be unsuitable for the AHP method because it can result in zero or infinite values in the normalized data, which is not acceptable for an AHP analysis (Vafaei et al., 2016). Thus, considering N8 for a comparative study involving AHP is meaningless.

### The second limitation and recommendation

Our literature analysis shows that the suitable normalization methods for some well-known MCDM techniques are yet to be amply explored. For instance, there were only 9% of the studies in Table 1 aimed to identify the ideal normalization methods for AHP. There are indeed some MCDM techniques which have completely been omitted in the previous comparative studies. For example, the suitability of the default normalization method for the ARAS, MOORA or α-Discounting technique (Smarandache, 2015) remains unexamined. Future work, therefore, can overspread its investigation to these less explored or unexplored MCDM techniques. Moreover, Chatterjee and Chakraborty (2014) claimed that different normalization techniques could yield distinct results, and therefore any investigation proposing the optimal normalization method for an MCDM technique must be welcomed. It is because the outcome of such an investigation would enable the decision-makers to swiftly make the rightest choice of normalization method before applying an MCDM technique, thus minimizing the bias in the results. Also, future researchers can consider synthesizing the results from different normalization methods (as presented in Table 1) to obtain a more compromised final solution (Wen et al., 2020).

### The third limitation and recommendation

Similar to the first limitation, no single study has simultaneously used all the eleven metrics identified in Table 3 to select the most appropriate normalization methods. Most of the existing studies used a limited set of metrics. Indeed, there are studies which merely used one metric, e.g., (Chakraborty and Yeh, 2009; Liao et al., 2012; Vafaei et al., 2018; Jafaryeganeh et al., 2020). It has to be reminded that using a limited set of metrics may cause inconclusive result issues. For example, Vafaei et al. (2016) concluded that N3 is the most suitable method for AHP after comparing five normalization methods based on two metrics. Four years later, the same team of researchers repeated a similar investigation (Vafaei et al., 2020), but the results favor N2 over N3. Such contradicting results hint that the set of metrics used can affect the final decision made on the available normalization methods. It is because, unlike the earlier study, the researchers used five metrics to decide the most suitable method in their latter study. Thus, future works should consider using a more comprehensive set of comparison metrics to ensure the conclusiveness of the results is not questionable.

### The fourth limitation and recommendation

Another limitation detected *via* this review relates to the RCI metric. Despite its popularity, this comparison metric appears to be unfitting for an MCDM problem with a larger decision matrix or when the comparison entails a large number of normalization methods (Aytekin, 2021). The computation of RCI immediately grows into a complex undertaking in such situations. Therefore, researchers can consider proposing a revised RCI with a simpler computational algorithm so that future comparative studies can utilize this metric, regardless of the size of the decision matrix or the number of normalization methods to compare.

### The fifth limitation and recommendation

As discussed earlier, the metrics in Table 3 are meant for determining the suitability of the normalization methods based on the result consistency or usability perspective. It is surprising that the suitability of the normalization methods has not been compared from the accuracy perspective. Accuracy refers to the degree of closeness of the result produced by a method over the true value (Trajkovic, 2008). Therefore, future comparative studies should consider using some accuracy metrics, such as mean square error, together with other metrics listed in Table 3, to grant a more inclusive and valid result.

## Conclusion

This study has gathered and reviewed articles testing the suitability of multiple normalization methods over an MCDM technique. The study has traced ten normalization methods that can potentially be used for solving an MCDM problem. In addition, eleven metrics were identified, and these metrics can be used to measure the suitability of normalization methods for an MCDM technique. Our study has also disclosed TOPSIS as the most popular MCDM technique examined for its suitable normalization methods. More importantly, the study has uncovered five shortcomings in the current literature and potential future works to address them were recommended.

From the literature perspective, this study can be considered the first endeavor to track and review past studies comparing the suitability of various normalization methods over an MCDM technique. Meanwhile, from the practical contribution perspective, the study conveys a clear message to the decision-makers that they must carefully choose the most suitable normalization method prior to employing an MCDM technique, so that a convincing solution can be reached for the problem at hand.

Nevertheless, our study has two limitations. The first limitation relates to the types of normalization methods considered in this study. It must be noted that the focus of our review was narrowed to dimensionless type of normalization methods. It is because these methods have the merit of allowing a logical aggregation of the values across both the benefit and cost criteria. The non-dimensionless methods, e.g., z-score and decimal normalization, were excluded and not reported in our study. However, future study may encapsulate all forms of normalization methods; hence, a more comprehensive list of methods can be presented for the benefit of decision-makers.

On the other hand, the second limitation of this study lies in the source used to locate the articles needed for the comparison. To be exact, the articles compared in this study were merely traced using Google Scholar since it is considered the largest search engine for academic papers. Future work, therefore, may extend its search for the relevant articles from more metadata services, e.g., Crossref and Ei Compendex, to deliver a more inclusive review.

## Author contributions

The author confirms being the sole contributor of this work and has approved it for publication.

## Funding

This work was supported by the Ministry of Higher Education (Malaysia) (Project code-RACER/1/2019/STG06/UMS/1).

## Conflict of interest

The author declares that the research was conducted in the absence of any commercial or financial relationships that could be construed as a potential conflict of interest.

## Publisher's note

All claims expressed in this article are solely those of the authors and do not necessarily represent those of their affiliated organizations, or those of the publisher, the editors and the reviewers. Any product that may be evaluated in this article, or claim that may be made by its manufacturer, is not guaranteed or endorsed by the publisher.
